# Exploring Active Compounds and Mechanisms of Angong Niuhuang Wan on Ischemic Stroke Based on Network Pharmacology and Molecular Docking

**DOI:** 10.1155/2022/2443615

**Published:** 2022-03-27

**Authors:** Yasu Zhang, Xiaomin Liu, Junzi Long, Xue Cheng, Xinyu Wang, Xiaodong Feng

**Affiliations:** School of Rehabilitation Medicine, Henan University of Chinese Medicine, Zhengzhou, Henan 450046, China

## Abstract

**Methods:**

The chemical ingredients of ANW were retrieved from TCMSP, TCMID, and literature. We predicted the potential targets of active ingredients by PubChem, Swiss Target Prediction, and STITCH databases. The targets related to ischemic stroke were retrieved using GeneCards, DisGeNET, DrugBank, TTD, and GEO databases. Subsequently, Venn diagrams were used to identify common targets of active ingredients and ischemic stroke. Protein-protein interaction (PPI) network was structured with STRING platform and Cytoscape 3.8.2. Gene ontology (GO) enrichment and Kyoto Encyclopedia of Genes and Genomes (KEGG) pathway analyses of key targets were performed in the Metascape database. Finally, molecular docking was conducted by AutoDock Tools and PyMOL software.

**Results:**

A total of 2391 targets were identified for 230 active ingredients of ANW, and 1386 of them overlapped with ischemic stroke targets. The key active ingredients were mainly quercetin, *β*-estradiol, berberine, wogonin, and *β*-sitosterol, and the key targets were also identified, including IL-6, AKT1, MAPK3, PIK3CA, and TNF. The biological process (BP) results indicated that ANW may have therapeutic effects through response oxidative stress, inflammatory response, cellular response to lipid, and response to nutrient levels. Furthermore, the ingredients of ANW were predicted to have therapeutic effects on ischemic stroke via the HIF-1 signaling pathway, FoxO signaling pathway, chemokine signaling pathway, fluid shear stress and atherosclerosis, and neurotrophin signaling pathway. The molecular docking results all showed that the core ingredients were strong binding activity with the core targets.

**Conclusion:**

In conclusion, the bioinformatics and pharmacological results reveal that counteracting oxidative stress, suppressing inflammation, inhibiting the development of AS, and even protecting neurological function are critical pathways for ANW in the treatment of ischemic stroke. These results may help to elucidate the mechanism of ANW on ischemic stroke for experimental studies and clinical applications.

## 1. Introduction

Ischemic stroke is a common cerebrovascular event due to an abrupt cerebral artery occlusion, resulting in insufficient perfusion, which then causes edema, inflammation, and necrosis of the affected tissue and severely damages to neurological function. The World Health Organization reports that ischemic stroke is the main cause of death and long-term disability in the world, which causes a tremendous psychological and financial burden on patients [1]. However, the pathological process of ischemic stroke involves multiple aspects, including energy metabolism disorder, oxidative stress, inflammation, and neuronal damage, and there is no sovereign remedy [2, 3]. Therefore, it is significantly important to explore drugs or active ingredients with multiple targets for the treatment of cerebral ischemia.

Notably, many of the Chinese herbs have been proven to produce therapeutic effects on ischemic stroke in clinical research [4]. As a famous Chinese herbal formula, Angong Niuhuang Wan (ANW) is widely used in clinical practice for the treatment of ischemic stroke, which contains 11 herbs, including *Moschus*, *Realgar*, *Curcumae Radix*, *Borneolum*, *Scutellariae Radix*, *Coptidis Rhizoma*, *Gardeniae Fructus*, *Bovis Calculus*, *Bubali Cornu*, *Margarita,* and *Cinnabaris.* Studies indicated that ANW had effect on reducing infarct size, protecting the integrity of the blood-brain barrier (BBB), improving antioxidant capacity, and inhibiting inflammation injury to produce neuroprotection; furthermore, it may improve the development of early atherosclerosis (AS) by suppressing inflammation [5–7]. However, the pharmacological effects of ANW on ischemic stroke have still not been elucidated.

**Figure 1:**
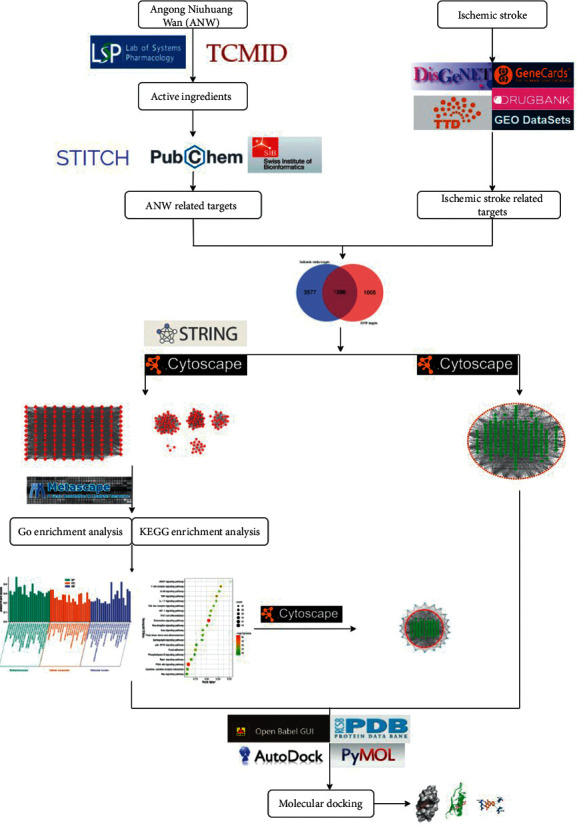
The workflow diagram of the study.

In this study, we aim to elucidate the possible mechanism of ANW on ischemic stroke and reveal the interaction between ANW, target, and ischemic stroke from a holistic perspective through a network pharmacology approach. The workflow diagram of the study is presented in Figure 1.

## 2. Material and Methods

### 2.1. Screening of Active Ingredients in ANW

The effective ingredients of ANW were searched through TCMSP (https://tcmspw.com/tcmsp.php) [8], TCMID (http://www.megabionet.org/tcmia/), and literature. The active compounds were screened for oral bioavailability (OB), drug-likeness (DL), and blood-brain barrier permeability (BBB) prediction. The selection of OB, DL, and BBB referred to the recommendations of the TCMSP database. Therefore, we finally screened the compounds with OB ≥ 0.2, DL ≥ 0.1, and BBB ≥ -0.3, which were considered as parameters for selecting potentially pharmacological ingredients [9–11], in addition, the ingredients with high content or pharmacological effects searched from literature and TCMID that did not contain the above parameters, which were also included in the further analysis. Besides, the threshold values were considered based on the following points: firstly, extracting more useful information from fewer compounds; secondly, maintaining concordance with the proven pharmacological data.

### 2.2. Prediction of Potential Targets of ANW

We retrieved SMILES number or 3D structure of each ingredient from the PubChem database (https://pubchem.ncbi.nlm.nih.gov/) and TCMID and inputted them into the PubChem, Swiss Target Prediction (http://www.swisstargetprediction.ch/) [12], and STITCH (http://stitch.embl.de/) database to obtain potential targets of bioactive ingredients. The target was further standardized in UniProtKB database (http://www.uniprot.org) [13].

### 2.3. Candidate Targets Collection of Ischemic Stroke

The disease targets correlated with “cerebral ischemic stroke” and “cerebral infarction” were identified through GeneCards (https://www.genecards.org/), DisGeNET (http://disgenet.org/), DrugBank (https://go.drugbank.com/), GEO (https://www.ncbi.nlm.nih.gov/geo/), and TTD (http://db.idrblab.net/ttd/) [14]. After deleting the duplicate targets of ischemic stroke, Venny 2.1 (http://bioinfogp.cnb.csic.Es/tools/venny/index.html) was used to identify common potential targets between ischemic stroke and the active ingredients of ANW.

### 2.4. Protein-Protein Interaction Network Construction and Analysis

Protein-protein interaction (PPI) network was constructed through the STRING database (https://string-db.org/) [15] with a confidence score >0.7. And topology analysis was performed by Cytoscape software. The key targets were sorted and screened according to the value of degree, betweenness centrality, and closeness centrality of the topological analysis results [16]. In addition, we screened important functional modules in PPI networks with the Cytoscape plugin MCODE.

### 2.5. Functional Enrichment and Pathways Analysis

The Gene ontology (GO) including biological process (BP), molecular function (MF), and cellular component (CC), and Kyoto Encyclopedia of Genes and Genomes (KEGG) pathway enrichment analyses were conducted using the Metascape database (https://metascape.org) [17]. The statistical significance threshold was set at the cutoff values of *P* < 0.01. In addition, the bioinformatics platform (http://www.bioinformatics.com.cn/) was used to visualize GO and KEGG enrichment analysis with the bubble charts.

### 2.6. Construction of Active Ingredients-Targets-Pathway Network

An ingredients-targets-network was constructed by Cytoscape software. The key active ingredients of ANW were sorted and screened according to the value of degree, betweenness centrality, and closeness centrality based on topological analysis.

### 2.7. Molecular Docking

The 3D structures of candidate ingredients were obtained from PubChem, which were transformed by Open Babel Toolkit (version 2.4.1) into a mol2 file format. The 3D structures of the core target were downloaded from the PDB database (http://www.rcsb.org/). The AutoDockTool 1.5.6 was used to add hydrogen and optimize protein structure for molecular docking after removing water and original ligands.

## 3. Results

### 3.1. Active Ingredients of ANW

A total of 230 active ingredients were obtained through the database after eliminating duplicates. These active ingredients were mainly derived from *Borneolum* (16 ingredients), *Bovis Calculus* (19 ingredients), *Coptidis Rhizoma* (17 ingredients), *Moschus* (32 ingredients), *Bubali Cornu* (22 ingredients), *Realgar* (3 ingredients), *Curcumae Radix* (44 ingredients), *Margarita* (16 ingredients), *Gardeniae Fructus* (22 ingredients), *Cinnabaris* (2 ingredients), and *Scutellariae Radix* (37 ingredients). Detailed active ingredients of ANW are shown in Table 1.

### 3.2. Protein-Protein Interaction Network Analysis

**Figure 2:**
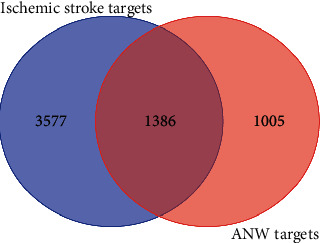
Venn diagram of ANW and ischemic stroke common targets.

**Figure 3:**
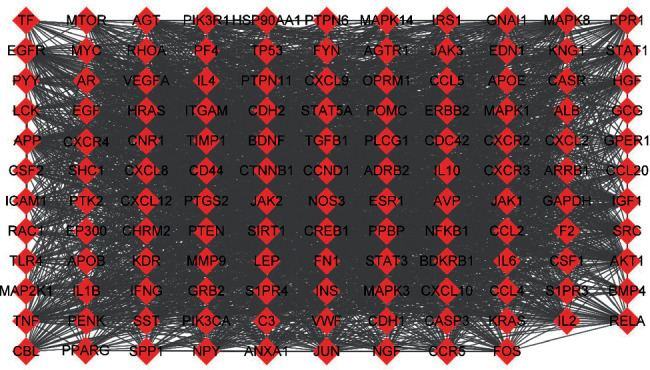
Protein-protein interaction network of core targets.

**Figure 4:**
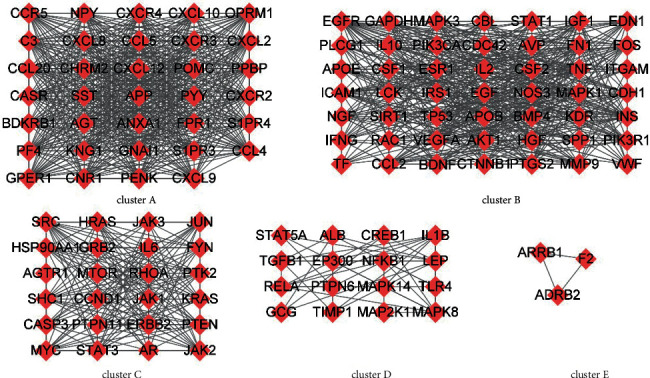
Subnetwork of targets PPI network.

A total of 4963 potential targets were obtained of ischemic stroke, and 1386 common targets were obtained after intersecting with 2391 potential targets of the active ingredients (Figure 2). The topological results of 1386 targets were obtained 130 significant targets according to the degree, betweenness centrality, and closeness centrality. The PPI network included 130 nodes and 2946 edges, among which 25 genes were more relevant to the ischemic stroke according to the MalaCards database (https://www.malacards.org/) [18], so they were identified as key targets (Figure 3, Table 2). MCODE has screened 5 functional modules according to the 130 targets (Figure 4). The biological functions of the subnetwork are shown in Table 3. The BP analysis revealed that the subnetworks were mainly associated with inflammatory response, response to lipid, neuroapoptosis, and development.

### 3.3. Construction of Active Ingredients-Targets Network

**Figure 5:**
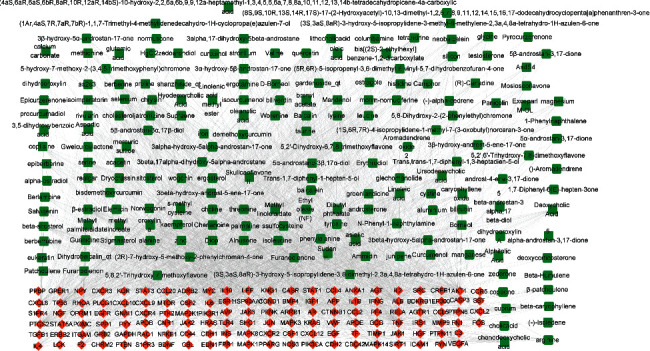
The active ingredients-targets network. Green represents active ingredients, and red represents the potential targets.

As shown in Figure 5, we constructed a network of active ingredients-targets using Cytoscape software (version 3.8.0). The active ingredients-targets network contained 310 nodes (including 180 ingredients and 130 genes) and 2110 edges. The top 20 active ingredients were screened by topology analysis (Table 4).

### 3.4. GO Enrichment Analysis

**Figure 6:**
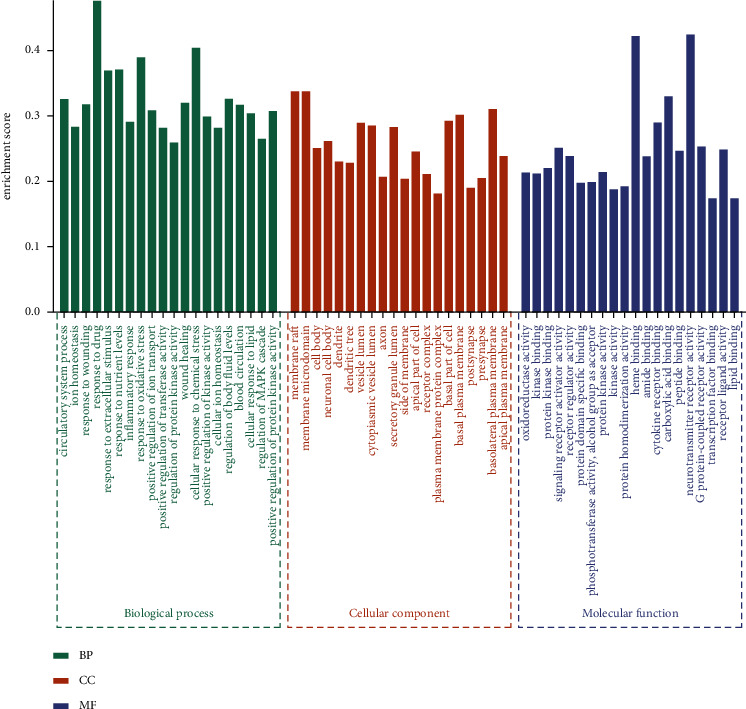
The GO enrichment analysis of 130 targets.

GO enrichment results include 296 BP terms, 99 MF terms, and 92 CC terms. The key items of BP mainly included response to oxidative stress, inflammatory response, cellular response to lipid, and response to nutrient levels. The main results of MF included oxidoreductase activity, cytokine receptor binding, lipid binding, and neurotransmitter receptor activity, and CC mainly included neuronal cell body, dendritic tree, axon, and postsynapse. We individually selected top 20 remarkably enriched terms in BP, MF, and CC classification as presented in Figure 6.

### 3.5. KEGG Pathway Enrichment Analysis and Ingredients-Targets Pathway Network Construction

**Figure 7:**
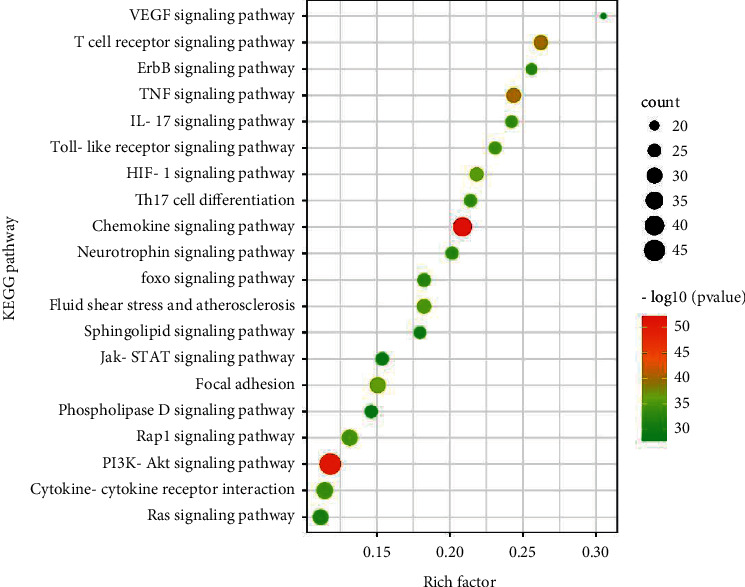
The KEGG enrichment analysis of 130 targets.

**Figure 8:**
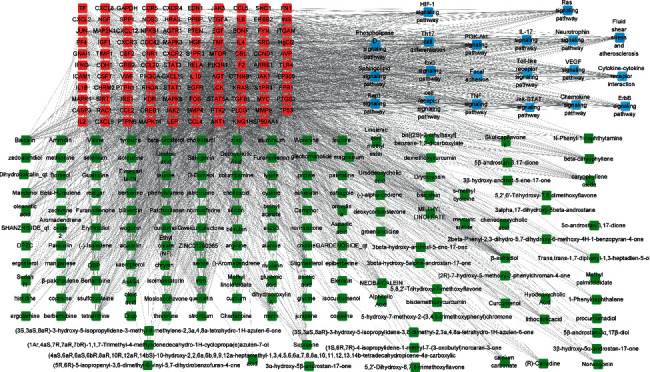
An ingredients-targets pathway network (green represents active ingredients, red represents potential targets, and blue represents the pathway).

KEGG pathway enrichment analysis may elaborate the mechanism of ANW on ischemic stroke. 139 signal pathways were obtained based on the 130 core targets. After removing pathways associated with cancer and unrelated to disease, the main results of KEGG pathways included the HIF-1 signaling pathway, FoxO signaling pathway, chemokine signaling pathway, fluid shear stress and atherosclerosis, and neurotrophin signaling pathway. 20 significantly enriched pathways were selected as shown in Figure 7. An ingredients-targets pathway network was built involving pathways, targets, and corresponding ingredients to further elucidate the molecular biological process of ANW for cerebral ischemic stroke (Figure 8). A total of 292 nodes (163 ingredients, 109 targets, and 20 pathways) and 2285 edges were obtained.

### 3.6. Docking Results Analysis

**Figure 9:**
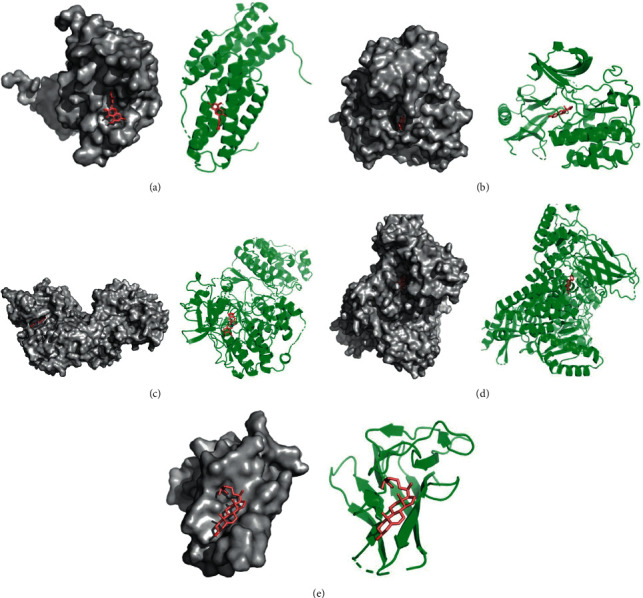
(a) Action mode of quercetin with target IL-6. (b) Action mode of *β*-estradiol with target AKT1. (c) Action mode of berberine with target MAPK3. (d) Action mode of wogonin with target PIK3CA. (e) Action mode of *β*-sitosterol with target TNF.

We selected the core targets, including IL-6, AKT1, MAPK3, PIK3CA, and TNF for molecular docking with the quercetin, *β*-estradiol, berberine, wogonin, and *β*-sitosterol. The results suggested that the 5 key ingredients all had a strong affinity with IL-6, AKT1, MAPK3, PIK3CA, and TNF, and the results of the docking were visualized by PyMOL software (Table 5, Figure 9).

## 4. Discussion

Stroke is classified as ischemic or hemorrhagic. Cerebral hemorrhage and cerebral ischemia have the possibility to cause serious inflammatory response, cerebral edema, and neurological deficits [19, 20]. The studies found that ANW reduced brain edema and intracranial pressure in cerebral ischemia and cerebral hemorrhage by regulating the expression of MMP-9 and AQP4 which were closely related to the formation of brain edema and the disruption of the BBB [21, 22]; in addition, it was able to exert neuroprotective function by reducing the inflammatory response and inhibiting oxidative stress and neurotoxicity in brain tissue of cerebral ischemia and cerebral hemorrhage [23, 24]. At present, the incidence of cerebral ischemia is far higher than cerebral hemorrhage; therefore, the paper is focused on the mechanism of Angong Niuhuang Wan in the treatment of cerebral ischemia. Network analysis increases the understanding of multiple mechanisms of drug action. Systems pharmacology may provide new avenues for drug discovery in complex diseases. Thus, network pharmacology may be helpful in excavating the potential mechanism of ANW for ischemic stroke.

The results of pharmaceutical ingredient analyses and molecular docking showed that the main ingredients quercetin, *β*-estradiol, berberine, and *β*-sitosterol showed strong binding activity to the IL-6, AKT1, MAPK3, PIK3CA of the core targets. IL-6 is a pleiotropic cytokine that plays a crucial role in host defense [25]. However, trans-signaling of IL-6 induces vascular endothelial cells to express and release the pro-inflammatory chemokine MCP-1, which is mediated through the JAK/STAT3 and PI3K/AKT pathways [26]. The studies found that administration of *β*-estradiol from *Moschus* reversed neuronal damage by regulating the JAK-STAT3 pathway and protected neurons from acidosis-mediated neurotoxicity and ischemic cerebral injury, thus promoting remodel and repair after brain injury [27, 28]. Liao et al. [29] demonstrated that *β*-sitosterol inhibited the secretion of inflammatory factors such as TNF-*α*, IL-1*β*, IL-6 to suppress the inflammatory response. TNF is a versatile pro-inflammatory cytokine involved in all stages of ischemic stroke. The study confirmed that quercetin from *Coptidis Rhizoma* and *Gardeniae Fructus* attenuated TNF-induced inflammation by suppressing the NF-*κ*B pathway [30]. MAPK is involved in inflammatory and apoptotic processes in cerebral ischemia-reperfusion injury. Studies had shown that quercetin inhibited inflammation and regulated JNK and ERK signaling pathways to produce antiapoptosis, thereby improving ischemic brain injury [31, 32].

AKT1, as a threonine protein kinases, is an important regulator of the AKT-mTOR signaling pathway that controls the tempo of newborn neurons during adult neurogenesis. PIK3CA is involved in the cell signaling of various growth factors. Yan et al. [33] demonstrated that activation of the PI3K/Akt/mTOR pathway inhibited oxidative stress-related neuronal autophagy and exerted neuroprotective functions. The research showed that berberine can reduce the apoptosis of striatum and mitochondrial through regulating PI3K/Akt signaling pathway and reducing intracellular ROS levels to exert neuroprotective effects [34, 35].

According to the results of KEGG enrichment analysis, ANW is considered to affect important pathways that are closely related to the pathogenesis of ischemic stroke, including HIF-1 signaling pathway, FoxO signaling pathway, chemokine signaling pathway, fluid shear stress and atherosclerosis, and neurotrophin signaling pathway. The results of GO enrichment were also closely related to response to oxidative stress, inflammatory response, cellular response to lipid, and response to nutrient levels. Furthermore, the BP analysis revealed that the subnetworks were mainly associated with inflammatory response, response to lipid, neuroapoptosis, and development.

HIF-1*α* is a primary modulator of cellular and systemic homeostatic reactions to hypoxia. Evidence showed that HIF-1 facilitated the transcription of various prosurvival proteins engaged in energy metabolism, angiogenesis, and neurogenesis, exerting a neuroprotective effect against ischemic stroke in ischemic conditions [36]. Research showed that estradiol facilitated neurogenesis in rats after stroke, possibly via increasing HIF-1*α* and VEGF protein expression [37]. The FoxO family of transcription factors is a critical regulator of cellular stress responses and facilitated the antioxidant defense of cells. Akt and p38MAPK are known stress-responsive kinases targeted to FoxO and are involved in the regulation of FoxO activity [38]. Zhang et al. [39] found that regulation of PI3K/Akt/FoxO-3a signaling pathway facilitated the proliferation of neural stem/progenitor cells and reduced ischemia-reperfusion injury. The inflammatory immune reaction needs leukocytes to be recruited to the site of inflammation. Chemokines are critical in protecting the host response by providing directional cues for cellular transport. Research confirmed that ANW downregulated the expression of chemokine receptors CCR2, CXCR3, and cell adhesion molecules in the arterial vasculature and alleviated the development of atherosclerosis by suppressing inflammation [7].

Atherosclerosis is a major cause of stroke onset or recurrence, and blood flow-induced shear stress has become an essential characteristic of atherosclerosis. The fluid resistance exerted on the vessel wall is mechanically translated into biochemical signals that lead to alter vascular behavior. Therefore, the maintenance of physiological laminar shear stress is essential for normal vascular function [40]. Quercetin alleviates vascular calcification by suppressing oxidative stress and mitochondrial division [41]. Moreover, Fan et al. [42] stated that ANW suppressed the development of atherosclerosis by regulating immune homeostasis and suppressing chronic inflammation. Neurotrophins have been proved to control survival, development, and function of neurons in the central nervous system. Studies asserted that quercetin and berberine alleviated neuronal apoptosis of ischemic stroke in the rat by activating the BDNF-TrkB-PI3K/Akt signaling pathway to increase the expression of BDNF [34, 43]. This suggests a potential application of neurotrophins in the therapy of ischemic stroke.

This research based on a pharmacological network explored the potential mechanisms of ANW for the treatment of ischemic stroke. The findings highlighted the improvement of the inflammatory response, immune defense, and neuroprotection of ANW against ischemic stroke. Our results were consistent with published studies that upregulation of HIF-1 signaling pathway, FoxO signaling pathway, and neurotrophin signaling pathway and downregulation of chemokine signaling pathway had positive effects on cerebral ischemia [42, 44–47]. In addition, we also provided some potential targets for treating ischemic stroke, which would contribute to the exploitation of new therapeutic strategies.

## 5. Conclusion

In conclusion, the bioinformatics and pharmacological results reveal that counteracting oxidative stress, suppressing inflammation, inhibiting the development of AS, and even protecting neurological function are critical pathways for ANW in the treatment of ischemic stroke. These results may help to elucidate the mechanism of ANW on ischemic stroke for experimental studies and clinical applications.

## Figures and Tables

**Table 1:** Information of the candidate active ingredients of ANW.

Herb	Active ingredients
*Bovis Calculus*	Oleanolic acid, cherianoine, CLR, bilirubin, methyl(4R)-4-[(3R,5S,7S,8R,9S,10S,12S,13R,14S,17R)-3,7,12-trihydroxy-10,13-dimethyl- 2,3,4,5,6,7,8,9,11,12,14,15,16,17-tetradecahydro-1H-cyclopenta[a]phenanthren-17-yl]pentanoate, methyl desoxycholate, chenodeoxycholic acid, deoxycholic acid, ZINC01280365, biliverdin, cholic acid, choline, deoxycorticosterone, ergosterol, ergotamine, glycocholic acid, lithocholic acid, ursodeoxycholic acid, hyodeoxycholic acid
*Coptidis Rhizoma*	Berberine, columbamine, fagarine, berberrubine, DPEC(5,8-dihydroxy-2-(2-phenylethyl)chromone), epiberberine, groenlandicine, (R)-canadine, berlambine, magnograndiolide, palmatine, coptisine, tetrandrine, Worenine, Pycnamine, jatrorrhizine, quercetin
*Scutellariae Radix*	Acacetin, wogonin, (2R)-7-hydroxy-5-methoxy-2-phenylchroman-4-one, *β*-patchoulene, baicalein, 5,8,2′-Trihydroxy-7-methoxyflavone, dihydrobaicalin_qt
	Salvigenin, 5,2′,6′-Trihydroxy-7,8-dimethoxyflavone, dihydrooroxylin A, skullcapflavone II, oroxylin a, panicolin, DIHYDROOROXYLIN(2beta-Phenyl-2,3- dihydro-5,7-dihydroxy-6-methoxy-4h-1-benzopyran-4-one), beta-sitosterol, sitosterol, norwogonin, 5,2′-dihydroxy-6,7,8-trimethoxyflavone, (-)-alpha-cedrene, linoleic acid, stigmasterol, dibutyl phthalate, coptisine, bis[(2S)-2-ethylhexyl] benzene-1,2-dicarboxylate, supraene, methyl palmitelaidate, methyl linolelaidate, Diop, epiberberine, patchoulene, 13-tetradecenyl acetate, moslosooflavone, 11,13-eicosadienoic acid, methyl ester, linolenic acid methyl ester, rivularin, neobaicalein, baicalin
*Bubali Cornu*	Calcium carbonate, eukeratin, ssulfocysteine, serine, isoleucine, glutamic acid, phenylalanine, histidine, cholesterol, cysteine, proline, lysine, tyrosine, arginine
	Ethanolamine, aspartic acid, glycine, alanine, methionine, threonine, guanidine derivatives, guanidine
*Moschus*	*β*-Estradiol, 3,5-dihydroxybenzoic acid, 3alpha,17-dihydroxy-5beta-androstane, 3alpha-hydroxy-5alpha-androstan-17-one, 3beta,17alpha-dihydroxy-5alpha-androstane, 3beta-hydroxy-5alpha-androstan-17-one, 3beta-hydroxy-androst-5-ene-17-one, 3*α*-hydroxy-5*β*-androstan-17-one, testosterone, allantoin, serine
	3*β*-Hydroxy-5*α*-androstan-17-one, 3*β*-hydroxy-androst-5-ene-17-one, 5 alpha-androstan-3,17-dione, 5beta-androstan-3 alpha,17beta-diol, 5*α*-androstan- 3,17-dione, 5*α*-androstane-3*β*,17*α*-diol, 5*β*-androstan-3,17-dione, 5*β*-androstan-3*α*,17*α*-diol, 5*β*-androstan-3*α*,17*β*-diol, alpha-estradiol, androst-4,6-diene-3,17-dione, androst-4-ene-3,17-dione, androsterone, cholesterol, decamine, estragole, morin, n-nornuciferine, normuscone, s-methyl cysteine, aspartic acid
*Cinnabaris*	Mercuric sulfide, HgCl2
*Gardeniae Fructus*	(4aS,6aR,6aS,6bR,8aR,10R,12aR,14bS)-10-Hydroxy-2,2,6a,6 b,9,9,12a-heptamethyl-1,3,4,5,6,6a,7,8,8a,10,11,12,13,14b-tetradecahydropicene-4a-carboxylic acid
	Ammidin, sudan III, linoleic acid, oleanolic acid, beta-sitosterol, stigmasterol, oleic acid, mandenol, supraene, methyl linoleate, methyl vaccinate, isoimperatorin
	Exceparl M-OL, chrysin, ethyl oleate (NF), 5-hydroxy-7-methoxy-2-(3,4,5-trimethoxyphenyl)chromone, PANA(N-Phenyl-1-naphthylamine), gardenoside_qt, quercetin, shanzhiside_qt, kaempferol
*Margarita*	Aluminium, calcium carbonate, cuprum, iron, manganese, silicon, zinc, magnesium, strontium, alanine, aspartic acid, leucine, serine, taurine, selenium, valine
*Borneolum*	Oleanolic acid, caryophyllene oxide, dipterocarpol, asiatic acid, bornyl acetate, beta-caryophyllene, borneol, isocembrol, D-borneol, erythrodiol, beta-humulene
	Oleanolic acid-28-O-beta-D-glucopyranoside, dryocrassin, camphor, elemicin, alphitolic acid
*Realgar*	Realgar, as2s3, As4S4
*Curcumae Radix*	Furanodienon, linoleic acid, beta-sitosterol, sitosterol, dibutyl phthalate, oleic acid, calarene, copaene, ()-aromadendrene, aromadendrene oxide 2, alnusone
	(1Ar,4aS,7R,7aR,7bR)-1,1,7-Trimethyl-4-methylidene decahydro-1h-cyclopropa(e)azulen-7-olTrans-1,7-diphenyl-1-hepten-5-ol, Junipene, ()-ledene, (4aR,5R,8 R, 8aR)-5,8-dihydroxy-3,5,8a-trimethyl-6,7,8,9-tetrahydro-4ah-benzo[f]benzofuran-4-one, curcumol, epicurzerenone, germacrone-4,5-epoxide, glechomanolide, furanodienone, isospathulenol, patchoulene, 1-phenylnaphthalene, pyrocurzerenone, trans,trans-1,7-diphenyl-1,3-heptadien-5-ol, zederone, bisdemethoxycurcumin, 1,7-diphenyl-6(E)-hepten-3one, calarenepoxide, caryophyllene oxide, (1S,3aR,4R,8aS)-7-isopropyl-1,4-dimethyl- 2,3,3a,5,6,8a- hexahydroazulene-1,4-diol, Isocurcumenol, (1S,6R,7R)-4-isopropylidene-1-methyl-7-(3-oxobutyl)norcaran-3-one, (5R,6 R)-5-isopropenyl-3,6-dimethyl-6-vinyl- 5,7-dihydrobenzofuran-4-one, (-)-isoledene, gweicurculactone, curcumenol, (3S,3aS,8aR)-3-hydroxy-5-isopropylidene-3-methyl-8-methylene-2,3a,4,8a- tetrahydro-1h-azulen-6-one, zedoarondiol, procurcumadiol, (3S,3aS,8aR)-3-hydroxy-5-isopropylidene-3,8-dimethyl-2,3a,4,8a-tetrahydro- 1h-azulen-6-one, 3-octadecenoic acid, demethoxycurcumin

**Table 2:** The information of the core targets.

Gene	Degree	Betweenness centrality
IL-6	91	0.030325537
AKT1	81	0.018339264
CXCL12	73	0.018125697
MAPK3	68	0.009734912
CXCR4	66	0.015168905
PIK3CA	65	0.012205642
TNF	61	0.00919471
AGT	59	0.008882338
MMP9	53	0.006325729
IL1B	51	0.0066625
ALB	51	0.007447992
PPBP	45	0.003930831
PF4	42	0.002706756
BDNF	40	0.003798139
NOS3	39	0.003217693
TLR4	38	0.002216187
AGTR1	37	0.004279922
CREB1	33	0.0019083
F2	32	0.002232586
CASP3	31	0.000874129
APOB	29	0.002920764
SIRT1	28	0.000753076
APOE	25	0.001338523
VWF	25	0.001087882
AVP	22	0.000773649

**Table 3:** The biological functions of subnetworks.

MCODE	GO	Description
A	GO:0006954	Inflammatory response
A	GO:0070098	Chemokine-mediated signaling pathway
A	GO:0006874	Cellular calcium ion homeostasis
B	GO:0070997	Neuron death
B	GO:0050900	Leukocyte migration
B	GO:0001568	Blood vessel development
C	GO:0007169	Transmembrane receptor protein tyrosine kinase signaling pathway
C	GO:0022407	Regulation of cell-cell adhesion
C	GO:0061564	Axon development
D	GO:1901652	Response to peptide
D	GO:0071396	Cellular response to lipid
D	GO:0002521	Leukocyte differentiation
E	GO:0008277	Regulation of *G* protein-coupled receptor signaling pathway
E	GO:0033674	Positive regulation of kinase activity
E	GO:0051347	Positive regulation of transferase activity

**Table 4:** List of core ingredients in the top 20.

Active components	Herbs	Degree	Betweenness centrality
Quercetin	Coptidis Rhizoma, Gardeniae Fructus	54	0.056874662
*β*-estradiol	Moschus	51	0.078777286
Tyrosine	Bubali Cornu	44	0.045643145
Berberine	Coptidis Rhizoma	40	0.018811059
Wogonin	Scutellariae Radix	39	0.021360805
Beta-sitosterol	Scutellariae Radix, Gardeniae Fructus, Curcumae Radix	37	0.019391773
Baicalein	Scutellariae Radix	36	0.016063119
Tetrandrine	Coptidis Rhizoma	35	0.013666927
chrysin	Gardeniae Fructus	34	0.013739355
Baicalin	Scutellariae Radix	32	0.008324522
Acacetin	Scutellariae Radix	31	0.013057
Oroxylin a	Scutellariae Radix	31	0.022607
Kaempferol	Gardeniae Fructus	31	0.009087
Demethoxycurcumin	Curcumae Radix	30	0.014592
Stigmasterol	Scutellariae Radix, Gardeniae Fructus	29	0.017506
Oleanolic acid	Bovis Calculus, Gardeniae Fructus, Borneolum	28	0.008257
Serine	Bubali Cornu, Moschus, Margarita	28	0.006694
Linoleic acid	Scutellariae Radix, Gardeniae Fructus, Curcumae Radix	27	0.010942
Oleic acid	Gardeniae Fructus, Curcumae Radix	26	0.012292
Ammidin	Gardeniae Fructus	26	0.011124

**Table 5:** Docking results of core active ingredients with core targets (kcal/mol).

Ingredients	IL-6	AKT1	MAPK3	PIK3CA	TNF
Quercetin	−5.68	−7.41	−6.39	−5.96	−6.39
*β*-Estradiol	−6.18	−9.05	−8.44	−8.28	−7.28
Berberine	−7.02	−8.69	−7.83	−8.8	−6.27
Wogonin	−5.37	−7.82	−6.79	−7.4	−6.62
*β*-Sitosterol	−6.54	−10.34	−8.54	−8.38	−7.65

## Data Availability

All data used to support the findings of this study are included within the article.
